# A Systematic Review of Trajectories of Clinically Relevant Distress Amongst Adults with Cancer: Course and Predictors

**DOI:** 10.1007/s10880-024-10011-x

**Published:** 2024-05-05

**Authors:** Leah Curran, Alison Mahoney, Bradley Hastings

**Affiliations:** 1 Health@Business Research Network, School of Management and Governance, University of New South Wales, High Street, Kensington, Australia; 2 The Kinghorn Cancer Centre, St Vincent’s Hospital Network, 370 Victoria Street, Darlinghurst, Sydney, NSW 2010 Australia; 3 Clinical Research Unit for Anxiety and Depression, St Vincent’s Hospital Network, 390 Victoria Street, Darlinghurst, Australia; 4 School of Psychiatry, University of New South Wales, High Street, Kensington, Australia

**Keywords:** Cancer, Distress trajectory, Predictors, Systematic review

## Abstract

To improve interventions for people with cancer who experience clinically relevant distress, it is important to understand how distress evolves over time and why. This review synthesizes the literature on trajectories of distress in adult patients with cancer. Databases were searched for longitudinal studies using a validated clinical tool to group patients into distress trajectories. Twelve studies were identified reporting trajectories of depression, anxiety, adjustment disorder or post-traumatic stress disorder. Heterogeneity between studies was high, including the timing of baseline assessments and follow-up intervals. Up to 1 in 5 people experienced persistent depression or anxiety. Eight studies examined predictors of trajectories; the most consistent predictor was physical symptoms or functioning. Due to study methodology and heterogeneity, limited conclusions could be drawn about why distress is maintained or emerges for some patients. Future research should use valid clinical measures and assess theoretically driven predictors amendable to interventions.

**Supplementary Information:**

The online version contains supplementary material available at 10.1007/s10880-024-10011-x.

## Introduction

Unsurprisingly, a diagnosis of cancer is often associated with psychological distress, such as symptoms of depression, anxiety, traumatic stress, fear of cancer progression or recurrence (FCR), or death anxiety. While longitudinal studies suggest that rates of distress generally improve over time, a substantial proportion of patients continue to experience clinically relevant distress (Calman et al., 2021; Henry et al., 2019; Krebber et al., 2014; Savard & Ivers, 2013). For mental health clinicians, understanding patients’ likely distress trajectory, specifically the timepoints at which distress is likely to emerge and whether the distress is likely to remain clinically relevant, is critical for the design and implementation of mental health and systemic interventions.

There are several gaps in the existing literature about *how* and *why* distress evolves amongst cancer patients. Firstly, longitudinal research has mainly reported prevalence data (Niedzwiedz et al., 2019), which conflates distress trajectories across different cancer cohorts and is not informative about how distress trajectories evolve for individual patients. For instance, there is evidence that distress increases longitudinally amongst younger patients and those with cancers that are more likely to recur, such as ovarian or oesophageal cancers (Liu et al., 2022; Starreveld et al., 2018; Watts et al., 2015). Additionally, there is scant longitudinal research regarding how distress evolves for individuals with advanced disease receiving novel therapies, who are likely at greater risk for psychological distress (Thewes et al., 2017). For example, amongst patients with advanced melanoma who achieved remission on immunotherapy, 64% had clinical levels of anxiety or depression at one point during the following year despite having no active disease (Rogiers et al., 2020). However, the distress trajectories and factors associated with recovered or persistent distress could not be ascertained by the data.

Secondly, longitudinal studies that group patients into distress trajectories vary in method. Some have used clinical cut-off scores and assessed individuals longitudinally at two time points (e.g. Linden et al., 2015), resulting in four clinically meaningful trajectories of distress: (1) non-cases (not meeting clinical criteria at either time point), (2) recovered (clinically significant distress at the first timepoint only), (3) persistent (meeting clinical criteria at both time points), and 4) emerging (clinically significant distress at the second timepoint only). In longitudinal studies conducted over more than two timepoints a fluctuating trajectory of distress may also be evident where a person meets clinical criteria at some, but not all, assessments (e.g., Mols et al., 2018). However, the majority of longitudinal studies of distress trajectories have used statistical methods, such as latent class growth analysis, to group participants into trajectories rather than utilising pre-determined clinical cut-off scores. Consequently, the number of trajectories identified, and the participants assigned to each trajectory, will differ according to the method used (e.g., Custers et al., 2020). Also, statistical analyses identify patterns in samples, so if the average outcome score is low, a statistically derived “persistently high” trajectory may include patients below a clinically meaningful threshold (e.g., Stanton et al., 2015). Since distress trajectories identified by statistical methods are difficult to interpret clinically, they have limited utility in informing clinical questions of how and why distress trajectories differ for individual patients.

Thirdly, it is unclear *why* some people are more likely to experience persistent or emerging distress over time. Theorists have suggested that intrapersonal and interpersonal constructs, such as metacognitions, cognitive appraisals, coping styles, physical symptom severity, social support, and the relationship with health care providers, may be important in understanding the evolution of distress and adjustment (Curran et al., 2017; Edmondson, 2014; Fardell et al., 2016; Kangas & Gross, 2020). However, the role of these constructs across the illness trajectory and within subgroups of patients is not clearly understood. For instance, a recent systematic review concluded that the only consistent psychological predictors of clinically relevant distress longitudinally are initial distress and neuroticism (Cook et al., 2018). However, this finding does not elucidate why some people had high distress scores at study entry or personality traits associated with experiencing more negative emotions. The reviewers called for better designed, theoretically informed longitudinal research to determine the psychological factors underpinning the aetiology and maintenance of cancer-related distress, which could then inform improvements in interventions. Separately, Kangas and Gross (2020) have called for further research to understand the trajectories of distress in a way that recognises cancer as a dynamic experience.

To address the above gaps, this study aims to summarize the literature regarding (1) the course of clinically relevant, individual trajectories of distress after a cancer diagnosis and (2) the psychological, sociodemographic and medical factors associated with different distress trajectories; and comment on how these findings relate to existing theories of cancer-related distress.

## Method

A systematic literature search was conducted following the PRISMA 2020 statement (Page et al., 2021). PsycINFO and Medline ALL were searched in December 2021 and updated in January 2023. Search terms related to clinical distress (depression, anxiety, trauma or stress related disorders), cancer *AND* longitudinal studies, were mapped to Medical Subject Headings (MeSH) and exploded where possible (see Supplementary Material for the full list of search items). The search was limited to peer-reviewed journals and articles in English, due to the unavailability of resources or funds to translate non-English articles. Reference lists of relevant articles were examined to identify further publications. Ethical approval was not required for this review of previously published data.

The first author (LC) inspected article titles and abstracts for the inclusion criteria: (1) written in English; (2) peer-reviewed; (3) adult patients with cancer; (4) mental health outcomes were assessed longitudinally with a validated clinically relevant measure and (5) results could be clustered according to clinical cut-off scores to identify distress trajectories. Articles were excluded if the sample (1) related to adult survivors of childhood cancer, (2) lacked statistical power to accurately determine the proportion of participants in each trajectory (power calculation in Supplementary Material), (3) only grouped patients into trajectories using statistical methods or (4) participants were enrolled in an intervention trial. A second reviewer (AM) independently examined 10% of article titles and abstracts, with disagreement resolved by consensus. The full text of retained articles were examined independently by both reviewers for further inclusion/exclusion with agreement reached by consensus.

The following data was obtained from the included studies by LC and fact-checked by AM: sample characteristics (sample size, age, gender, cancer type and stage, place of recruitment), time of study entry (T1), time since diagnosis, follow-up timepoints and the interval from T1, the proportion of patients in active treatment at follow-up, outcome measure used, trajectories identified, proportion of sample in each trajectory, and predictors of between group differences (if examined). When trajectories were not reported in the article, but data was available to calculate them, the proportion of patients in each trajectory was calculated from the completer sample. Results were tabulated according to the clinical outcome measured, and the number of assessments conducted longitudinally. Due to the heterogeneity of studies, a meta-analysis was not possible, and a narrative review was conducted.

The methodological quality of included studies was evaluated independently by LC and AM using four domains from the Quality in Prognosis Studies (QUIPS) tool (Hayden et al., 2013): study participation, attrition, outcome measurement and study confounding. A fifth domain, statistical analyses, was rated only if analyses of between group factors were conducted (some studies only reported descriptive statistics to calculate the proportion of the sample in each distress trajectory).

## Results

**Fig. 1:**
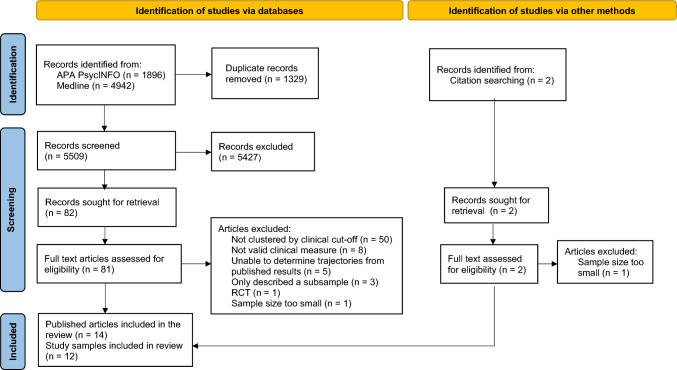
PRISMA flow diagram of systematic search results

Figure 1 outlines the process of article selection and reasons for exclusion. After removing duplicates, 5509 articles were identified. Inter-rater reliability (Cohen’s Kappa) of 10% of the articles was 0.73. Title and abstract review yielded 82 articles for full-text review and two articles were identified via the ancestry method. Independent review of the articles resulted in full consensus to retain 14 articles, describing 12 samples. Studies were excluded where the proportion of patients in each distress trajectory could not be calculated from the published data (e.g., Lopes et al., 2022; Sutton et al., 2022).

Table 1 provides the characteristics of the 12 study samples included in this review (N = 8566). Results are discussed according to the outcome measured. Also, the results are discussed according to the number of timepoints measured, as this impacts on the potential number of trajectories identified, and the proportion of patients assigned to each trajectory.

**Table 1:** Studies included in the systematic review

First author and year	Cancer type and stages	Country	Recruitment source	Age recruited and M (SD)	Proportion female	N^a^	T1 assessment	Months post-diagnosis at T1: M (SD)	FU interval(s) from T1 in months	% in active Tx at FU	Measure and clinical cut-off score	Trajectories and proportions^b^
*Depression studies: two assessment timepoints and four trajectories*												
Alfonsson (2016)	Breast All stages	Sweden	Breast Cancer Quality Register	25–94 60.6 (11.6)	100%	833	1–9 months after diagnosis	4(NR)	38	NR	HADS-D ≥ 8	80% non-cases, 9% recovered, 6% emerging, 5% persistent
Boyes (2013)	Mixed All stages	Australia	2 state-based cancer registries	18–80 NR^d^	42%	1125	6 months post diagnosis	NR	6	23%^e^	HADS-D ≥ 8	82% non-cases, 6% recovered, 6% emerging, 6% persistent
Hasegawa (2019)	Lymphoma or Multiple Myeloma All stages	Japan	Hospital inpatients intending to undergo chemotherapy	≥ 20 years NR^d^	47%	255	Pre-chemotherapy	NR	1	100%	PHQ-9 ≥ 10	67% non-cases, 15% recovered, 7% emerging, 10% persistent
Kim (2012, 2013, 2018)	Breast Not “secondary breast cancer”	South Korea	Hospital inpatients undergoing mastectomy	25–80 50.8 (9.7)	100%	247	Post-mastectomy	2.5(7.7)	12	NR	MINI (minor or major depression)	67% non-cases, 16% recovered, 10% emerging, 7% persistent
Linden 2015)	Mixed^f^ All stages	Canada	Provincial cancer clinic	“Legal age” 60.8 (12.6)	54%	334	Pre-treatment	NR	12	NR	PSSCAN subscale ≥ 8	64% non-cases, 11% recovered, 19% emerging, 4% persistent
Sullivan (2016)	Lung All stages	USA	Consortium of 7 research groups	≥ 21 years NR^d^	45%	1155	5 months post diagnosis	NR	7	NR	CES-D short form ≥ 4	55% non-cases, 14% recovered, 9% emerging, 22% persistent
*Depression studies: three or more assessment timepoints*												
Jansen (2018)	Head and neck All stages Treated with curative intent	United Kingdom	Multiple clinical centres	≥ 16 years NR^d^	23.4%	1217	Pre-treatment	NR	4, 12	NR	HADS-D ≥ 8	63% non-cases, 16% recovered, 7% persistent, 1% recurrent, 12% late emerging
Mols (2018)	Colorectal All stages	The Netherlands	Cancer registry	≥ 20 years 69.4 (9.5)	44.9%	1241	1 to 10 years post diagnosis	62.4(33.6)	12, 24, 36	NR	HADS-D ≥ 8	71% non-cases, 8% persistent, 21% fluctuating
*Anxiety studies: two assessment timepoints and four trajectories*												
Alfonsson (2016)	Breast All stages	Sweden	Breast Cancer Quality Register	25–94 60.6 (11.6)	100%	833	1–9 months after diagnosis	4(NR)	38	NR	HADS-A ≥ 8	61% non-cases, 13% recovered, 11% emerging, 15% persistent
Boyes (2013)	Mixed^c^ All stages	Australia	2 state-based cancer registries	18–80 NR^d^	42%	1125	6 months post diagnosis	NR	6	23%^e^	HADS-A ≥ 8	70% non-cases, 8% recovered, 7% emerging, 14% persistent
Kim (2020)	Breast Stages 0–III Excluded if recurred	South Korea	2 cancer hospitals	≥ 18 years 46.4 (7.9)	100%	250	Pre-surgery	NR	12	NR	HADS-A ≥ 8	48% non-cases, 23% recovered, 9% emerging, 21% persistent
Linden (2015)	Mixed^f^ All stages	Canada	Provincial cancer clinic	“Legal age” 60.8 (12.6)	54%	334	Pre-treatment	NR	12	NR	PSSCAN subscale ≥ 8	55% non-cases, 17% recovered, 10% emerging, 18% persistent
*Anxiety studies: three or more assessment timepoints*												
Mols (2018)	Colorectal All stages	The Netherlands	Cancer registry	≥ 18 years 69.4 (9.5)	44.9%	1241	1 to 10 years post diagnosis	62.4(33.6)	12, 24, 36	NR	HADS-A ≥ 8	68% non-cases, 10% persistent, 22% fluctuating
*Combined anxiety and depression*												
Boyes (2013)	Mixed^c^ All stages	Australia	2 state-based cancer registries	18–80 NR^d^	42%	1125	6 months post diagnosis	NR	6	23%^e^	HADS-A ≥ 8 AND HADS-D ≥ 8	87% non-cases, 4% recovered, 5% emerging, 4% persistent
*Adjustment disorder (AD) or other mental disorder (MD)*												
Wijnhoven (2022)	Breast Stages I–III Treated with curative intent	The Netherlands	3 hospitals	≥ 18 years 57.8 (9.3)	100%	293	within 5 years of completing primary treatment	33.1(16.1)	3, 6, 12	NR	AD = HADS-T 11–14 MD = HADS-T ≥ 15	54% non-cases, 1% persistent AD, 7% persistent OMD, 38% fluctuating
*PTSD*												
Smith (2011)	Non Hodgkin’s Lymphoma All stages	USA	2 cancer centres	≥ 19 years 62.4 (12.4)	52%	557	≥ 2 years post diagnosis	124.8(85.2)	60	NR	PCl-C ≥ 3 symptoms	90% non-cases, 3% recovered, 4% emerging, 3% persistent
Vin-Raviv (2013)	Breast Stages I–III	USA	Multiple clinical sites	≥ 20 years NR^d^	100%	1059	2–3 months post diagnosis	NR	2, 4	NR	IES ≥ 24	T2: 72% non-cases, 12% recovered, 5% emerging, 11% persistent T3: 71% non-cases, 9% recovered and sustained, 4% late recovery, 4% emerged but then resolved.7% persistent, 2% fluctuating, 1% emerged and sustained, 2% late onset

*FU* follow-up, *M* mean, *NR* not recorded, *OMD* other mental disorder, *SD* Standard Deviation, *Tx* treatment

Instruments: *CES-D* Centre for Epidemiological Studies Depression Scale-8 item short-form, *HADS-A* Hospital Anxiety and Depression Scale – anxiety subscale, *HADS-D* Hospital Anxiety and Depression Scale – depression subscale, *HADS-T* Hospital Anxiety and Depression Scale – total score, *IES* Impact of Event Scale, *MINI* Mini International Neuropsychology Interview, *PCl-C* Post traumatic Stress Disorder Checklist- Civilian Version, *PHQ-7* Patient Health Questionnaire, *PSSCAN* Psychosocial Screen for Cancer

^a^Subsample that completed all longitudinal assessments; ^b^proportions calculated from reported data; ^c^mixed sample included prostate 24%, breast 19%, colorectal 17%, melanoma 16%, lung 12%, non-Hodgkin’s lymphoma or leukemia 8%, or head and neck 4%; ^d^age reported as a categorical variable or by trajectory rather than sample mean; ^e^15% received “passive” treatment (hormonal therapy, bone marrow or stem cell transplant or immunotherapy) and 8% received “active” treatment (chemotherapy or radiotherapy ± “passive” treatment) in the month before the T2 assessment; ^f^mixed sample included breast 42%, genitourinary 18%, gastrointestinal 9%, sarcoma 5%, head and neck 4%, lung 4%, melanoma 4%, lymphoma 3%, leukemia 1% or other 10%

### Depression Studies with Two Assessment Timepoints

Six studies assessed depression over two timepoints to identify four trajectories: non-cases, recovered, emerging and persistent (Alfonsson et al., 2016; Boyes et al., 2013; Hasegawa et al., 2019; Kim et al., 2012; Linden et al., 2015; Sullivan et al., 2016). Studies involved breast (Alfonsson et al., 2016; Kim et al., 2012), lung (Sullivan et al., 2016), or mixed cancer diagnoses (Boyes et al., 2013; Linden et al., 2015) or malignant Lymphoma or Multiple Myeloma (Hasegawa et al., 2019). In the non-breast cancer studies, male and female patients were equally represented. One study excluded patients with “secondary” disease (Kim et al., 2012) and three studies excluded terminally ill patients or those deemed unsuitable or too unwell by their physician to participate (Boyes et al., 2013; Hasegawa et al., 2019; Sullivan et al., 2016). Only three studies reported patients’ mean age, and participants were typically in their 50 s and 60 s (Alfonsson et al., 2016; Kim et al., 2012; Linden et al., 2015). Further analysis of one study sample (Kim et al., 2012) produced two additional publications (Kim et al., 2013, 2018).

Caseness was usually determined by cut-off scores on self-report measures such as the depression subscale of the Hospital Anxiety and Depression Scale (HADS-D; Zigmond & Snaith, 1983), the depression subscale of the Psychosocial Screen for Cancer (PSSCAN; Linden et al., 2005), the Patient Health Questionnaire (Kroenke et al., 2001), and the Centre for Epidemiological Studies Depression Scale– short-form (Turvey et al., 1999). A structured diagnostic interview was only used in one sample and participants were classified as cases if they met criteria for minor or major depression (Kim et al., 2012). Assessment points varied considerably. Two studies assessed depression prior to treatment but the samples were followed up one month or 12 months later (Hasegawa et al.; Linden et al., 2015). One study assessed depression post-surgery and 12 months later (Kim et al., 2012). Three studies assessed depression 4–6 months post-diagnosis (on average) and followed up six (Boyes et al., 2013), seven (Sullivan et al., 2016), or 38 months later (Alfonsson et al., 2016). Most studies did not report whether patients were in active treatment throughout the course of the assessment period or had completed treatment.

In terms of depression trajectories, about half of patients with lung cancer were classified as non-cases (Sullivan et al., 2016), compared to two thirds of participants in other newly diagnosed cancer groups (Hasegawa et al., 2019; Kim et al., 2012; Linden et al., 2015). The highest rates of non-cases were reported in two studies that recruited patients 4–6 months post-diagnosis (80–82%; Alfonsson et al., 2016; Boyes et al., 2013). The proportion of recovered cases varied from 6 to 16% and was lowest in the sample that had low rates of depression at baseline (Alfonsson et al., 2016). The sample of patients with lung cancer had the highest rate of persistent depression (22%) measured over a seven-month period (Sullivan et al., 2016). The remaining studies reporting rates of persistent depression at 4–10%, measured over intervals ranging from 1–36 months. Emerging depression was most common in a mixed sample of newly diagnosed patients assessed before treatment and 12 months later (19%; Linden et al., 2015). The remaining studies reported emerging depression rates of 6–10%.

Group comparisons were conducted on three study samples to identify predictors associated with depression trajectories (Table 2). Amongst patients with newly diagnosed haematological malignancies, non-cases were more likely to be physically active compared to those with persistent depression (Hasegawa et al., 2019). No other demographic or medical variables were differentially associated with trajectories. Similarly, amongst newly diagnosed patients with breast cancer, recovery from depression was associated with greater improvements in general health, emotional, social and role functioning, fatigue, and insomnia over the 12-month follow-up period, compared to persistent cases (Kim et al., 2013). Conversely, persistent or emerging depression was associated with larger decrements in general health, and emotional and role functioning, and worsening physical symptoms compared to non-cases. Persistent depression was also associated with financial difficulties, personal or family history of depression, more metastatic axillary lymph nodes, larger tumour size, and specific genotypes compared to non-cases (Kim et al., 2012, 2013). However, age, education, time since diagnosis or tumour stage were not associated with depression trajectories.

**Table 2:** Studies reporting predictors of trajectories

Outcome	Depression					Anxiety			PTSD	AD
First author and year(s)	Hasegawa (2019)	Kim (2012, 2013, 2018)	Jansen (2018)	Linden (2015)	Mols (2018)	Kim (2020)	Linden (2015)	Mols (2018)	Vin-Raviv (2013)	Wijnhoven (2022)
*Demographic variables*										
Age	NS	NS	✓	✓	✓	NS	✓	✓	✓	✓
Gender	NS	NA	NS	–	NS	NA	–	✓	NA	NA
Marital/partner status	NS	–	✓	–	NS	NS	–	NS	–	NS
Education	NS	NS	✓	–	✓	NS	–	✓	–	NS
Income	–	–	✓	NS	–	NS	NS	–	–	–
Occupation/employment status	NS	–	–	–	–	NS	–	–	–	–
Race	–	–	–	–	–	–	–	–	✓	
*Disease variables*										
Tumour stage, size or genotype; number of lymph nodes	NS	✓	✓	NS	✓	NS	NS	✓	NS	–
Treatment type	–	NS	✓	–	NS	NS	–	NS	NS	–
Time since diagnosis	–	NS	–	–	NS	–	–	NS	–	NS
Comorbidities	–	–	✓	–	✓	–	–	✓	–	–
General health/functioning	✓	✓	–	–	–	–	–	–	–	✓
Physical symptoms such as fatigue, nausea, pain	–	✓	–	–	–	✓	–	–	–	–
*Lifestyle variables*										
Alcohol consumption	–	–	✓	–	–	–	–	–	–	–
Smoking	–	–	✓	–	–	–	–	–	–	–
*Psychological variables*										
Baseline depression	–	✓	–	✓	–	–	✓	–	–	–
Baseline anxiety	–	–	–	✓	–	–	✓	–	–	–
History of mood disorder/counselling	–	✓	–	–	–	–	–	–	–	NS
Family history of mood disorder	–	✓	–	–	–	–	–	–	–	–
Illness intrusiveness	–	–	–	✓	–	–	✓	–	–	–
Social support	–	–	–	NS	–	–	NS	–	–	✓
Optimism	–	–	–	–	–	–	-	–	–	✓
Neuroticism	–	–	–	–	-	–	–	–	–	✓

*NS* not significant, *NA* not applicable (all female); ✓ significantly different by trajectory; – not examined in analysis of predictors of trajectories

In contrast, younger age was associated with persistent depression in a study of people with various cancer diagnoses (Linden et al., 2015). Higher illness intrusiveness (i.e., the perceived impact of illness on functioning) was associated with persistent depression, although illness intrusiveness did not differentiate between persistent, recovered, or emerging groups. Persistent depression was also associated with higher baseline anxiety and depression scores. However, post-hoc analysis showed that baseline anxiety and depression scores were equally high for persistent and recovered cases and equally low for non-cases and emerging cases, suggesting that baseline anxiety and depression scores had limited utility in predicting depression trajectories.

### Depression Studies with More Than Two Assessment Timepoints

Two studies assessed depression trajectories over three or more time points using a HADS-D score of > 8 to define caseness. Newly diagnosed patients with head and neck cancer, treated with curative intent, were assessed for depression before commencing treatment and 4 and 12 months later (Jansen et al., 2018). The number of patients still in active treatment was not reported. Five trajectories were identified: non-cases (63%), recovered (16%), persistent (7%), late emerging (12%), and recurrent (1%). Compared to non-cases and those who recovered, persistent, emerging or recurrent depression was associated with being single, widowed or divorced, lower education, lower income, later tumour stage at diagnosis, having chemotherapy, more co-morbidities, smoking and being a non-drinker or a hazardous drinker of alcohol. Age was associated with depression trajectories but was assessed as a categorical variable, making the results difficult to interpret.

Patients from a colorectal cancer registry who were one to ten years post-diagnosis were assessed yearly for depression over four years (Mols et al., 2018). Three depression trajectories were identified: non-cases (71%), persistent (8%) and fluctuating (21%). Those with persistent or fluctuating depression were more likely to have two or more comorbidities compared to non-cases. Fluctuating depression was also associated with older age, lower education, and stage IV disease (compared to stage III as assessed at study entry). Changes in disease status or treatment course were not assessed over time.

### Anxiety Studies with Two Assessment Timepoints

Four studies assessed clinically relevant anxiety over two timepoints. Caseness was defined by a cut-score of ≥ 8 on the anxiety subscale of the HADS (HADS-A) or PSSCAN (Alfonsson et al., 2016; Boyes et al., 2013; Kim et al., 2020; Linden et al., 2015). Two studies involved women with breast cancer: women with stage 0-III cancer were assessed pre-surgery and 12 months later (Kim et al., 2020), and women with all cancer stages were assessed within 9 months of diagnosis and 3 years later (Alfonsson et al., 2016). The other two anxiety studies involved people with mixed cancer diagnoses assessed 6 months post-diagnosis and 6 months later (Boyes et al., 2013), and assessed before treatment commenced and 12 months later (Linden et al., 2015). Boyes et al. (2013) reported that 8% of their sample were receiving chemotherapy or radiotherapy at follow-up, but the proportion of patients in active treatment was not reported in the other studies.

Despite differences in samples and assessment timepoints across studies, some patterns emerged. Studies that recruited patients before commencing treatment had lower proportions of “non-cases” (48%-55%) and higher proportions of recovered cases (17–23%; Kim et al., 2020; Linden et al., 2015) compared to the studies that recruited on average 4–6 months after diagnosis (61–70% non-cases and 8–13% recovered cases; Alfonsson et al., 2016; Boyes et al., 2013). The rate of emerging anxiety was about 10% and was lowest in the study that recruited patients 6 months after diagnosis (7%; Boyes et al., 2013). The rate of persistent anxiety ranged from 14 to 21%, and was highest in a sample of newly diagnosed women with breast cancer (Boyes et al., 2013; Kim et al., 2020).

Two of these studies examined predictors of anxiety trajectories (Table 2). Persistent anxiety was associated with younger age and higher baseline anxiety or depression severity compared to non-cases; and recovered anxiety was associated with lower baseline anxiety compared to persistent cases (Linden et al., 2015). Persistent and emerging anxiety was also associated with more pain, breast and arm symptoms at follow-up compared to non-cases (Kim et al., 2020).

### Anxiety Studies with More Than Two Assessment Timepoints

One study assessed patients with colorectal cancer for clinically relevant anxiety at diagnosis and yearly for three years (Mols et al., 2018). Using the HADS-A, patients were grouped into three trajectories: non-cases (68%), persistent (10%) and fluctuating (22%). As with depression, those with fluctuating or persistent anxiety were more likely to have two or more comorbidities compared to non-cases (Table 2). Fluctuating anxiety was also associated with being female, younger age, lower education, and stage IV disease (compared to stage I).

### Mixed Anxiety and Depression

One study assessed patients who scored above the clinical cut-off on both the depression and anxiety HADS subscales (Boyes et al., 2013). Not surprisingly, the proportion of patients in the clinical range was much smaller than for anxiety or depression alone (5% emerging, 4% persistent).

### Adjustment Disorder (AD)

One study examined AD (defined as marked distress not meeting criteria for another mental disorder) amongst patients with breast cancer who had completed treatment within the previous 5 years (Wijnhoven et al., 2022). Patients were assessed four times over 12 months using the HADS total score (HADS-T). Scores of 11–14 were defined as AD, and scores ≥ 15 were defined as “other mental disorder.” Participants were classified into 4 trajectories: non-cases (54%), fluctuating (38%), persistent “other mental disorder” (7%), and persistent AD (1%). Persistent or fluctuating trajectories (combined and compared to non-cases) were associated with younger age, more difficulties with daily activities, less social support than desired, lower optimism, and higher neuroticism.

### Post-Traumatic Stress Disorder (PTSD)

Two studies examined PTSD trajectories. Patients with Non-Hodgkin’s Lymphoma (NHL) were assessed for PTSD two years post-diagnosis and five years later (Smith et al., 2011). 10% of the sample were receiving active treatment at study entry but the proportion receiving active treatment at follow-up is not reported. Male and female participants were equally represented, and sample mean age was 62 years. Using the PTSD Checklist-Civilian Version (PCL-C; Weathers et al., 1993), those at least moderately bothered by one re-experiencing, three avoidance and two arousal symptoms were classified as PTSD cases. Most patients in this cohort were non-cases for PTSD (90%), with small proportions of recovered (3%), emerging (4%) and persistent (3%) cases reported. The authors examined predictors of PCL-C scores not PTSD trajectories.

In the second study, patients with stage I-III breast cancer were assessed for PTSD on three occasions: 2–3, 4 and 6 months after diagnosis (Vin-Raviv et al., 2013). Whether participants were actively receiving cancer treatment was not assessed. Caseness was determined by Impact of Events Scale score ≥ 24 (Horowitz et al., 1979). At 4 months post-diagnosis, four trajectories were identifiable from the published data: non-cases (72%), recovered (12%), persistent (11%), and emerging (5%). At 6 months post-diagnosis, eight trajectories were identifiable. Four trajectories described those who did not meet criteria for PTSD at 6 months: non-cases (71%), sustained recovery (9%), PTSD emerged then resolved (4%), and late recovery (4%). Four trajectories described patients who met criteria for PTSD at 6 months: persistent (7%), late onset (2%), resolved but re-emerged (2%), and emerged and sustained (1%). Non-cases were compared to those who met PTSD criteria at two or more consecutive assessments. No clinical variables distinguished these groups, but non-cases were more likely to be aged over 50 and be White or Hispanic rather than Black or Asian.

## FCR and Death Anxiety

No studies meeting our inclusion criteria examined trajectories of FCR or death anxiety.

### Risk of Bias Assessment

Risk of bias assessment results are presented in Table 3 (see Supplementary Material for more detail). Inter-rater agreement was 84%. No studies were rated as having a low risk of bias across all domains. Of clinical importance, four studies were rated high on risk of bias for study participation due to low recruitment rates (38–51%; Boyes et al., 2013; Kim et al., 2020; Sullivan et al., 2016; Wijnhoven et al., 2022), while six studies were rated high on risk of bias for attrition, due to low completer rates (53–64%; Alfonsson et al., 2016; Jansen et al., 2018; Kim et al., 2020; Wijnhoven et al., 2022), non-completers were more likely to be anxious or depressed,^31^ or key characteristics of non-completers were not described (Linden et al., 2015; Wijnhoven et al., 2022). In terms of outcome measurement, the window for baseline data collection post-diagnosis was wide in five studies (Alfonsson et al., 2016; Boyes et al., 2013; Mols et al., 2018; Smith et al., 2011; Wijnhoven et al., 2022). None of the studies examining why the trajectory groups differed (Hasegawa et al., 2019; Jansen et al., 2018; Kim et al., 2012, 2020; Linden et al., 2015; Mols et al., 2018; Vin-Raviv et al., 2013; Wijnhoven et al., 2022) were informed by a theoretical model.

**Table 3:** Quality assessment of included studies based on the QUIPS tool (Hayden et al., 2013)

Lead author and publication date	Population and participation	Attrition	Outcome measurement	Confounders	Statistical analysis/reporting
Alfonsson et al. (2016)	M	H	H	L	–
Boyes et al. (2013)	H	M	M	L	–
Hasegawa et al. (2019)	L	L	M	L	M
Jansen et al. (2018)	M	H	L	M	M
Kim et al. (2020)	H	H	L	L	M
Kim et al. (2012, 2013, 2018)	L	M	L	M	L
Linden et al. (2015)	M	H	L	H	L
Mols et al. (2018)	L	H	H	M	L
Smith et al. (2011)	M	M	M	M	–
Sullivan et al. (2016)	H	M	L	M	–
Vin-Raviv et al. (2013)	L	L	L	M	L
Wijnhoven et al. (2022)	H	H	M	M	L

*L* low risk of bias, *M* medium risk of bias, *H* high risk of bias, – not applicable

## Discussion

This is the first review to synthesise the current knowledge about longitudinal distress trajectories in patients with cancer. We identified 12 study samples assessing trajectories of depression (8), anxiety (5), PTSD (2) and AD (1). Unfortunately, due to heterogeneity between studies, we were unable to conduct a meta-analysis on the prevalence of trajectories or the predictors. Nevertheless, patterns did emerge in the findings which are discussed below.

### Depression

Findings suggest that depression trajectories may be related to cancer cohorts, with higher levels of persistent depression evident in the study of patients with lung cancer (Sullivan et al., 2016). These findings contrast with a meta-analysis reporting that the average point-prevalence of depression amongst patients with lung cancer was similar to other cancer groups (Krebber et al., 2014). A key limitation of the cross-sectional prevalence studies included in the meta-analysis is that they do not inform who continued to have depression over time. For lung cancer, high rates of persistent depression have been associated with stigma (Cataldo & Brodsky, 2013), poor sleep quality (He et al., 2022), impacts on physical functioning (Hopwood & Stephens, 2000), and diagnosis commonly occurring at a later stage (Schabath & Cote, 2019). Within this context, early screening for depression may be a more robust indicator of persistent distress amongst lung cancer patients and flag the need for psychological intervention, compared to other cancer groups.

Findings also indicate that future studies should take greater account of survivor bias. Rates of non-cases of depression were highest amongst the two studies that recruited from large cancer registries (Alfonsson et al., 2016; Boyes et al., 2013) and were therefore more likely to include patients who had completed their initial treatment. Conversely, rates of recovery from depression were lower when initial assessments were conducted some months after diagnosis (Alfonsson et al., 2016; Boyes et al., 2013) compared to those studies that recruited close to diagnosis (Jansen et al., 2018; Kim et al., 2012). These data suggest that future research should commence longitudinal studies closer to diagnosis to allow comparisons across studies and identify patients who recover, and the protective factors associated with their recovery.

No conclusions could be drawn from the data about when depression is likely to emerge after a cancer diagnosis. Rates of emerging depression were highest amongst newly diagnosed patients with mixed cancer types assessed before treatment and 12 months later (19%; Linden et al., 2015). The 12-month assessment point was chosen to coincide with patients completing their initial treatment regime and adjusting to survivorship. However, some patients were in the palliative phase and about 5% of patients died during the follow-up period. Given the heterogeneity in the sample and the long interval between assessment timepoints, future studies should assess more frequently to determine when clinically relevant depression emerged, and for which patients, so that screening efforts can be directed accordingly.

Regarding predictors of depression trajectories, findings indicate that greater emphasis is needed on understanding physical symptom severity and psychological factors as predictors of depression. Physical symptom severity as measured by performance status (Hasegawa et al., 2019), the impact of physical symptoms on functioning (Kim et al., 2012, 2013), or the presence of comorbidities (Jansen et al., 2018; Mols et al., 2018), was consistently associated with persistent depression. Demographic variables were not significant predictors (Jansen et al., 2018; Kim et al., 2013; Linden et al., 2015; Mols et al., 2018), and age inconsistently predicted depression trajectories (Hasegawa et al., 2019; Jansen et al., 2018; Linden et al., 2015). Clinical variables, such as time since diagnosis, treatment type, or tumour stage were also not significant predictors of persistent depression in most studies. Importantly, one study assessed changes in physical symptoms over time (Kim et al., 2013). Surprisingly, no studies examined whether being in active treatment or completing treatment explained distress trajectories. As adaptation to cancer evolves within a changing context of symptoms and functioning, contextual physical and treatment variables should also be assessed longitudinally and considered in future predictive models.

The two studies exploring psychological predictors of depression reported that a baseline or previous history of depression was associated with a persistent depression trajectory (Kim et al., 2012; Linden et al., 2015), suggesting that previous history should be included in early psychological screening processes. This also suggests that exploring the vulnerability factors that predisposed these individuals to depression is important in identifying psychological predictors amenable to treatment. For instance, theories suggest that habitual coping strategies and the quality of social support may impede or facilitate adjustment to cancer (Kangas & Gross, 2020) Separately, illness intrusiveness, or the subjective meaning and salience of symptoms, was argued to be a psychological predictor associated with persistent depression (Linden et al., 2015). The illness intrusiveness rating scale used in this study assesses the extent to which illness disrupts functioning in various quality-of-life domains (Devins et al., 2001), and may be a proxy for symptom severity. Consequently, more refined measures of illness meaning and salience are needed. Interestingly, there is evidence that related psychological constructs, such as illness representations and the impact of illness on self-schemas, are associated with depression amongst people with cancer (Carpenter et al., 2009; Richardson et al., 2017). These constructs are important to investigate in future research to identify psychological predictors of depression trajectories that may be amenable to interventions.

### Anxiety

As with depression, anxiety trajectories were related to assessment timepoints. Studies that recruited at least 6 months after diagnosis from cancer registries had the highest proportion of non-cases (Boyes et al., 2013; Mols et al., 2018) while studies that recruited before treatment commenced had the lowest rate of non-cases and higher rates of persistent and recovered anxiety (Kim et al., 2020; Linden et al., 2015). The early phase of adjustment to diagnosis and treatment is an especially anxious period for patients who are experiencing uncertainty about treatment efficacy and the possible impacts on their roles and relationships (Thewes et al., 2017). However, a substantial proportion of patients did adjust without any specific intervention.

Findings regarding the emerging anxiety trajectory highlight the importance of assessing patients at multiple timepoints to identify patients in need of clinical intervention. While emerging anxiety was the least common trajectory, around 1 in 10 people did develop anxiety over time, even amongst those recruited up to nine months after diagnosis (Alfonsson et al., 2016). The rate of emerging anxiety occurred similarly across studies, suggesting this phenomenon was related to aspects of the cancer experience that are common across cancer cohorts, such as ongoing uncertainty and fears about the cancer progressing or recurring (Curran et al., 2017).

In terms of predictors of anxiety trajectories, demographic variables, such as education, income and age, were not consistent predictors (Kim et al., 2020; Linden et al., 2015; Mols et al., 2018). As with depression, cancer symptoms, comorbidities and/or general health functioning were associated with persistent anxiety (Kim et al., 2020; Mols et al., 2018). Higher illness intrusiveness and physical symptoms were also associated with emerging anxiety (Kim et al., 2020; Linden et al., 2015). This accords with the Enduring Somatic Threat Model, based on Terror Management Theory, that posits that physical symptoms remind a person of their mortality and inflate anxiety (Edmondson, 2014). Consequently, it is important that future studies assess contextual physical factors longitudinally to understand the course of anxiety and ensure effective physical symptom management to improve mental health outcomes.

Only one study examined predictors of recovered anxiety, and reported that, compared to persistent anxiety, baseline anxiety was lower (Linden et al., 2015). However, this does not inform us about why these patients had lower anxiety at baseline. Given the paucity of research on the predictors of anxiety trajectories, theoretically informed research is needed to understand the vulnerability and protective factors that underpin anxiety trajectories.

#### PTSD

The two studies assessing PTSD trajectories also highlight a need for future studies to carefully consider assessment time-points (Smith et al., 2011; Vin-Raviv et al., 2013). Not surprisingly rates of persistent PTSD were lowest in the sample that included people on average 7 years post diagnosis (Smith et al., 2011). Neither of these studies examined predictors of trajectories specifically, although higher PTSD scores were associated with greater impacts of the cancer on appearance, life interference and worrying (Smith et al., 2011), and younger patients were more likely to meet criteria for PTSD on two consecutive longitudinal assessments (Vin-Raviv et al., 2013). Other possible predictors of persistent PTSD that are amenable to interventions warrant further investigation, such as a history of trauma or major life stressors prior to the cancer diagnosis (Silver-Aylaian & Cohen, 2001; Swartzman et al., 2017), avoidant coping (Jacobsen et al., 2002), greater illness uncertainty (Kuba et al., 2017), higher disease burden (Kuba et al., 2017; Shand et al., 2015), and lower social support (Jacobsen et al., 2002; Shand et al., 2015).

Since these studies, diagnostic criteria have changed so that PTSD is diagnosed only when there has been a sudden, catastrophic event in addition to the cancer diagnosis (such as a life-threatening haemorrhage), and the person experiences intrusive, hyperarousal and avoidance symptoms related to memories of that event (APA, 2013). Consequently, in the cancer context, where intrusions are generally related to *future-orientated* fears, PTSD should be rarely diagnosed; instead, patients with cancer who experience traumatic stress symptoms would likely meet criteria for AD (Kangas, 2013). Indeed, some researchers have argued that the classification of AD as a distinct stress disorder more appropriately describes the distress experienced in cancer settings and therefore may lead to improvements in research and interventions (Esser et al., 2019).

#### AD

Only one study examined trajectories of AD (Wijnhoven et al., 2022), so no meaningful conclusions can be drawn from our review of the literature to date. The study was limited to patients with breast cancer who had completed treatment given with curative intent. Only 1.4% met the study criteria for persistent AD, that is, marked but not high levels of distress on the HADS-T. The low case-rate for persistent AD may relate to assessment validity or that the study included patients that would be expected to be past the initial post-diagnosis adjustment phase. More studies are needed to understand the trajectory of AD from diagnosis.

### FCR or Death Anxiety

No studies examining trajectories of FCR met our inclusion criteria, principally because many studies of FCR have used statistical methods to group participants into trajectories rather than clinical cut-off scores. Investigating FCR is important because FCR is a pattern of worry, preoccupation and hypervigilance to body symptoms centred on fears about the cancer progressing or recurring, that can be distinguished from other anxiety disorders (Mutsaers et al., 2020). FCR is also likely to have a unique course and predictors with evidence that FCR persists over time, and may even increase in severity, especially for younger patients (Starreveld et al., 2018). Also, no studies of trajectories of death anxiety were reported. Death anxiety is important to consider as it is associated with, but distinct from other manifestations of anxiety, such as general anxiety, health anxiety or FCR (Curran et al., 2020; Menzies et al., 2021), and occurs across cancer cohorts, affecting people with metastatic or late-stage cancer (Lo et al., 2011; Neel et al., 2015) as well as people who have completed their cancer treatment and seem to be disease free (Cella & Tross, 1987; Lagerdahl et al., 2014). The lack of research on death anxiety may be due to there being no agreed definition of clinically relevant death anxiety, and the commonly used measures of death anxiety were developed for patients with incurable disease and their validity has not been tested in other cancer populations (Sharpe et al., 2018).

### Study Limitations

While our review of the literature on distress trajectories was comprehensive, there were some limitations. The review was not pre-registered. Also, the findings were limited to articles published in English. While it is possible that some important non-English publications may have been missed, this approach is a standard methodology for reviews of cancer-related themes (e.g. Arring et al., 2023; Hasson-Ohayon et al., 2022). Further, given that our review aimed to identify the clinical utility of research to date, we decided to use clinical terms in our search strategy, such as anxiety, depression and adjustment disorder, rather than a generic search term, such as distress. We expected that the course, predictors, and potential intervention targets of various clinically meaningful psychological states, such as anxiety or depression, would differ and would not be captured if these constructs were conflated into a broader category of distress. This may have meant that we failed to capture the diversity of people’s experiences of distress after a cancer diagnosis.

Furthermore, the generalisability of our findings to the broader cancer population may be limited due to several considerations. Arguably, study quality criteria relating to rates of study participation and attrition are particularly important for indicating the clinical importance or meaningfulness of results because these criteria demonstrate how representative the study samples are of the general cancer population, and therefore how well results reflect clinical cohorts in naturalistic settings. Across studies, rates of participation and attrition were not robust, and studies typically excluded individuals with advanced cancers, those who were very unwell, and those with a previous cancer diagnosis. Bias due to attrition was only low in two studies, and individuals who did not complete follow-up assessments were generally more psychologically distressed and physical unwell at baseline compared to those who completed all study measures (Alfonsson et al., 2016; Boyes et al., 2013; Mols et al., 2018). Given that poorer physical health status was associated with persistent depression or anxiety, it is likely that our findings underestimate the prevalence of clinically meaningful distress amongst individuals with cancer. Future longitudinal research should maximise recruitment inclusivity across all stages of cancer and systematically account for study attrition.

Additionally, consistent findings regarding distress trajectories were difficult to determine due to methodological heterogeneity. Studies varied by the number of follow-up assessments conducted and the number of trajectories identified, with possible implications for the proportion of patients assigned to each trajectory. Also, initial assessments ranged from close to diagnosis (Jansen et al., 2018), when distress may be expected to be higher, to years after diagnosis (Mols et al., 2018). Follow-up assessments were generally conducted at pre-determined study time points rather than at times that are clinically meaningful or personally relevant to cancer patients such as prior to surveillance scans, or around diagnosis anniversary dates. Also, whether patients were receiving active treatment was generally not assessed. Incomplete clinical data about the context in which assessments occurred limits the clinical utility and ecological validity of findings and precludes conclusions regarding the optimal time to assess psychological distress in routine care. Future longitudinal research should consider taking measurements from the point of diagnosis, followed by meaningful points along the cancer care pathway.

Furthermore, it is unclear whether the psychological assessments conducted in the reviewed studies were optimal. Only one sample completed a structured diagnostic interview (Kim et al., 2012, 2013, 2018), but minor and major depression were merged to categorise caseness, potentially confounding the findings. While the most frequently used self-report measure was the HADS, there has been debate regarding optimal cut-off values (Annunziata et al., 2020; Vodermaier et al., 2009), and concern over its use as a case-finding instrument (Mitchell, 2010). This highlights the need for foundational research to establish valid case-finding measures so that clinically meaningful research can be conducted.

Lastly, there was limited assessment of psychological variables predicting distress trajectories. Studies generally investigated demographic and medical variables with little evaluation of psychological constructs that may explain the aetiology or maintenance of distress and are responsive to psychotherapy. For instance, one interesting area of research is coping strategies and how they may impact on emotion regulation differently at various phases of diagnosis, treatment, survivorship, recurrence and end of life (cf. Kangas & Gross, 2020). Future longitudinal studies need to assess therapeutically relevant constructs informed by theoretical models of cancer-related distress.

## Conclusions

This study aimed to examine the course of clinically relevant, individual trajectories of distress after a cancer diagnosis and the psychological, sociodemographic and medical factors associated with different distress trajectories. The limited findings from this review suggest that depression screening efforts should be particularly directed at patients with lung cancer. Also, longitudinal approaches to screening are needed to detect emerging depression and anxiety, since up to 1 in 5 patients developed depression in the first year after diagnosis and about 1 in 10 developed clinically relevant anxiety (Kim et al., 2020; Linden et al., 2015). The review also highlighted that consistent assessment timepoints across studies are needed to establish a wider evidence base to inform screening efforts. Furthermore, since assessments conducted at diagnosis or during clinic visits capture distress that is understandable and often subsides, more finely grained assessments, such as utilising smart phone applications, would be useful in future studies to understand when high distress is likely to be sustained. This would ensure that interventions are utilised efficiently, do not pathologize understandable distress and allow natural adaptation to occur, while ensuring that unremitting distress is treated as early as possible.

Understanding why distress develops, resolves or continues is important clinically to elucidate the protective and maintaining factors underpinning distress trajectories, thereby providing intervention targets that can be tailored to individual trajectories. The review suggests that symptom burden was the most consistent predictor of persistent distress, highlighting that mental health interventions should be multi-disciplinary in their approach. Furthermore, prior history should be screened as a potential predictor of depression. Disappointingly, there was limited information about other psychosocial predictors that could guide interventions. Future research should be guided by theoretical models of cancer-related distress, such as cognitive processing and metacognitive approaches (Cook et al., 2015; Curran et al., 2017; Edmondson, 2014; Fardell et al., 2016; Kangas & Gross, 2020), that may identify targets for treatments.

## Supplementary Information

Below is the link to the electronic supplementary material.

Supplementary file1 (DOCX 40 KB)

## Data Availability

More detail about QUIPs assessment is available in Supplementary Materials.
