# Supplementary material for: A Systematic Review of Trajectories of Clinically Relevant Distress Amongst Adults with Cancer: Course and Predictors
Source: J Clin Psychol Med Settings. 2024 May 5;32(1):1–18. doi: 10.1007/s10880-024-10011-x (PMC11914336; doi:10.1007/s10880-024-10011-x)
Supplement: Supplementary file 1 — Supplementary file1 (DOCX 40 KB) [file 10880_2024_10011_MOESM1_ESM.docx]

**Appendix A: Supplementary Information**

Table A1. Search terms for PsycINFO (searched on December 7, 2021 and updated January 27, 2023)

| 1. | neoplasm.mp. or exp Neoplasms/ |
| --- | --- |
| 2. | limit 1 to (peer reviewed journal and english language) |
| 3. | oncology.mp. or exp Oncology/ |
| 4. | limit 3 to (peer reviewed journal and english language) |
| 5. | cancer.mp. |
| 6. | limit 5 to (peer reviewed journal and english language) |
| 7. | h*ematology.mp. [mp=title, abstract, heading word, table of contents, key concepts, original title, tests & measures, mesh word] |
| 8. | limit 7 to (peer reviewed journal and english language) |
| 9. | "h*ematopoietic stem cell transplant".mp. [mp=title, abstract, heading word, table of contents, key concepts, original title, tests & measures, mesh word] |
| 10. | limit 9 to (peer reviewed journal and english language) |
| 11. | chemotherapy.mp. or exp Chemotherapy/ |
| 12. | limit 11 to (peer reviewed journal and english language) |
| 13. | radiotherapy.mp. |
| 14. | limit 13 to (peer reviewed journal and english language) |
| 15. | 2 or 4 or 6 or 8 or 10 or 12 or 14 |
| 16. | exp Death Anxiety/ or exp Health Anxiety/ or exp Anxiety Disorders/ or exp Anxiety/ or exp Generalized Anxiety Disorder/ or anxiety.mp. |
| 17. | limit 16 to (peer reviewed journal and english language) |
| 18. | exp "Depression (Emotion)"/ or exp Major Depression/ or exp Reactive Depression/ or exp Recurrent Depression/ or depression.mp. |
| 19. | limit 18 to (peer reviewed journal and english language) |
| 20. | "adjustment disorder".mp. or exp Adjustment Disorders/ |
| 21. | limit 20 to (peer reviewed journal and english language) |
| 22. | "traumatic stress".mp. or exp Posttraumatic Stress/ |
| 23. | limit 22 to (peer reviewed journal and english language) |
| 24. | "fear of cancer progression".mp. |
| 25. | limit 24 to (peer reviewed journal and english language) |
| 26. | "fear of cancer recurrence".mp. |
| 27. | limit 26 to (peer reviewed journal and english language) |
| 28. | hypochondriasis.mp. or exp Hypochondriasis/ |
| 29. | limit 28 to (peer reviewed journal and english language) |
| 30. | exp Somatoform Disorders/ or "somatic symptom disorder".mp. |
| 31. | limit 30 to (peer reviewed journal and english language) |
| 32. | trajector*.mp. |
| 33. | limit 32 to (peer reviewed journal and english language) |
| 34. | exp Longitudinal Studies/ or longitudinal.mp. |
| 35. | limit 34 to (peer reviewed journal and english language) |
| 36. | exp Prospective Studies/ or prospective.mp. |
| 37. | limit 36 to (peer reviewed journal and english language) |
| 38. | 33 or 35 or 37 |
| 39. | 17 or 19 or 21 or 23 or 25 or 27 or 29 or 31 |
| 40. | 15 and 38 and 39 |

Table A2. Search terms for MedlineALL (searched on December 7, 2021 and updated January 27, 2023)

| 1. | neoplasm.mp. or Neoplasms/ |
| --- | --- |
| 2. | limit 1 to english language |
| 3. | oncology.mp. or Medical Oncology/ or Radiation Oncology/ or Psycho-Oncology/ or Surgical Oncology/ |
| 4. | limit 3 to english language |
| 5. | cancer.mp. [mp=title, abstract, heading word, table of contents, key concepts, original title, tests & measures, mesh word] |
| 6. | limit 5 to english language |
| 7. | Hematology/ or h*ematology.mp. |
| 8. | limit 7 to english language |
| 9. | "h*ematopoietic stem cell transplant".mp. [mp=title, abstract, heading word, table of contents, key concepts, original title, tests & measures, mesh word] |
| 10. | limit 9 to english language |
| 11. | chemotherapy.mp. |
| 12. | limit 11 to english language |
| 13. | radiotherapy.mp. [mp=title, abstract, heading word, table of contents, key concepts, original title, tests & measures, mesh word] |
| 14. | limit 13 to english language |
| 15. | exp Anxiety/ or anxiety.mp. or exp Anxiety Disorders/ |
| 16. | limit 15 to english language |
| 17. | depression.mp. or exp Depression/ |
| 18. | limit 17 to english language |
| 19. | "adjustment disorder".mp. or exp Adjustment Disorders/ |
| 20. | limit 19 to english language |
| 21. | exp Stress Disorders, Post-Traumatic/ or "traumatic stress".mp. |
| 22. | limit 21 to english language |
| 23. | "fear of cancer progression".mp. |
| 24. | limit 23 to english language |
| 25. | "fear of cancer recurrence".mp. |
| 26. | limit 25 to english language |
| 27. | hypochondriasis.mp. or exp Hypochondriasis/ |
| 28. | limit 27 to english language |
| 29. | exp Somatoform Disorders/ or "somatic symptom disorder".mp. |
| 30. | limit 29 to english language |
| 31. | trajector*.mp. |
| 32. | limit 31 to english language |
| 33. | exp Longitudinal Studies/ or longitudinal.mp. |
| 34. | limit 33 to english language |
| 35. | exp Prospective Studies/ or prospective.mp. |
| 36. | limit 35 to english language |
| 37. | 2 or 4 or 6 or 8 or 10 or 12 or 14 |
| 38. | 16 or 18 or 20 or 22 or 24 or 26 or 28 or 30 |
| 39. | 32 or 34 or 36 |
| 40. | 37 and 38 and 39 |

Table A3. Quality assessment of included studies using the QUIPS tool (Hayden et al., 2013)

| Study – Lead Author | Alfonsson | Boyes | Hasegawa | Jansen | Kim 2020 | Kim 2018 | Linden | Mols | Smith | Sullivan | Vin-Raviv | Wijnhoven |
| --- | --- | --- | --- | --- | --- | --- | --- | --- | --- | --- | --- | --- |
| **1. Study population and participation** |  |  |  |  |  |  |  |  |  |  |  |  |
| i) Characteristics of source population adequately described | + | + | + | + | + | + | + | + | + | + | + | + |
| ii) Sampling frame and recruitment | + | + | + | + | + | + | + | + | + | + | + | +/- |
| iii) Recruitment period | + | - | + | + | + | + | - | + | +/- | + | + | - |
| iv) Place of recruitment | + | +/- | + | + | + | + | - | + | + | + | + | +/- |
| v) Inclusion and exclusion criteria | + | +/- | + | +/- | + | + | + | + | + | + | + | + |
| vi) Participation (≥70%, or the nonresponse was not selective) | - | - | + | - | - | + | +/- | +/- | +/- | - | + | - |
| vii) Baseline sample characteristics adequately described | + | + | + | +/- | + | + | +/- | + | +/- | + | +/- | +/- |
| **Study Participation Summary Score** | M | H | L | M | H | L | M | L | M | H | L | H |
| **2. Study Attrition** |  |  |  |  |  |  |  |  |  |  |  |  |
| i) Response rate (≥80%, or the nonresponse was not selective) | - | + | + | - | - | + | + | - | - | - | + | - |
| ii) Attempts to collect information on whose who dropped out | - | + | - | - | - | - | +/- | - | + | +/- | - | - |
| iii) Reasons for loss to follow-up provided | - | + | + | + | + | - | +/- | +/- | + | +/- | - | - |
| iv) Description of participants lost to follow-up | +/- | + | - | + | - | - | - | + | + | +/- | - | - |
| v) No important differences between completers & non-completers | - | - | + | - | ? | + | - | - | - | +/- | ? | ? |
| **Study Attrition Summary Score** | H | M | L | H | H | M | H | H | M | M | L | H |
| **3. Outcome measurement** |  |  |  |  |  |  |  |  |  |  |  |  |
| i) Clear definition of the outcome and follow-up period | +/- | + | + | + | + | + | + | + | + | + | + | + |
| ii) Reliable and valid measurement | + | + | + | + | + | + | + | + | + | + | + | +/- |
| iii) Timing of the measurement window comparable for all participants (within a month) | - | - | +/- | ? | ? | ? | ? | - | - | ? | ? | ? |
| **Outcome Measurement Summary Score** | H | M | M | L | L | L | L | H | M | L | L | M |
| **4. Study Confounding** |  |  |  |  |  |  |  |  |  |  |  |  |
| i) All potentially important confounders are measured* | + | + | + | +/- | + | + | - | + | +/- | +/- | +/- | +/- |
| ii) Valid and reliable measurement of confounders | + | + | + | + | + | + | + | + | + | +/- | + | - |
| iii) Method used for missing data | ? | ? | ? | ? | ? | ? | ? | ? | ? | ? | ? | ? |
| iv) Potential confounders accounted for in study design | - | - | - | +/- | - | - | - | +/- | +/- | + | +/- | +/- |
| v) Potential confounders accounted for in analysis | + | + | + | - | + | + | - | + | +/- | + | +/- | +/- |
| **Study Confounding Summary Score** | L | L | L | M | L | M | H | M | M | M | M | M |
| **5. Statistical analysis and reporting (studies analysing between group differences)** | | | | | | | | | | | |  |
| i) Sufficient presentation of data to assess adequacy of analysis |  |  | - | + | + | + | + | + |  |  | + | + |
| ii) Variables included in the statistical model are appropriate and based on a conceptual framework |  |  | - | - | - | - | - | - |  |  | +/- | - |
| ii) Selected statistical model is adequate for the study design |  |  | +/- | +/- | +/- | + | + | + |  |  | + | + |
| iii) Reporting of results |  |  | + | + | + | + | + | + |  |  | + | + |
| **Statistical Analysis and Reporting Summary Score** | | | M | M | M | L | L | L |  |  | L | L |

Legend: Issue adequately reported: + = yes, - = no, +/- = partial, ?= not reported;

L= low risk of bias, M= medium risk of bias, H= high risk of bias

*age, gender, cancer site, stage, treatment, time since diagnosis, and symptom severity or performance status

**Power calculations for sample size**

**A1.** Formula used to calculate the minimum sample size needed to estimate the proportion of adults who are non-cases within 90% confidence (Daniel, 1999)**:**

n = $\hat{p}$ $\times\hat{q}$ $\times\frac{Z(\alpha/z)}{E}$

where: $\hat{p}$ = proportion of the sample

$\hat{q}$ = 1-$\hat{p}$;

E = margin of error at 5%

$Z(\alpha/z)$= 1.645 for p = 0.05

Table A4. Sample size calculations for different mental health outcomes.

| **Outcome** | $\hat{\boldsymbol{p}}$ | **Source** | **N** |
| --- | --- | --- | --- |
| Depression | 0.82 | Boyes et al., 2013 | ≥ 160 |
| Anxiety | 0.7 | Boyes et al., 2013 | $\geq$ 228 |
| Mixed anxiety and depression | 0.5 | Default $\hat{p}$ value of 0.5 used as no reference in the literature | ≥ 271 |
| PTSD | 0.9 | Smith et al., 2011 | $\geq$ 98 |
| Fear of Cancer Recurrence or Progression | 0.5 | Default $\hat{p}$ value of 0.5 used as no reference in the literature | ≥ 271 |
| Death Anxiety | 0.5 | Default $\hat{p}$ value of 0.5 used as no reference in the literature | ≥ 271 |

**References:**

Boyes, A.W., Girgis, A., D'Este, C.A., Zucca, A.C., Lecathelinais, C., & Carey, M.L., 2013. Prevalence and predictors of the short-term trajectory of anxiety and depression in the first year after a cancer diagnosis: a population-based longitudinal study. J. Clin. Oncol. 31(21), 2724-2729.

Daniel, W.W., 1999. Biostatistics: a foundation for analysis in the health sciences. 7th ed. New York: John Wiley & Sons.

Smith, S.K., Zimmerman, S., Williams, C.S., Benecha, H., Abernethy, A.P., Mayer, D.K., Edwards, L.J., Ganz, P.A., 2011. Post-traumatic stress symptoms in long-term non-Hodgkin's lymphoma survivors: does time heal? J. Clin. Oncol. 29(34), 4526-4533.
